# Matrix-assisted laser desorption ionization mass spectrometry based quantitative analysis of cordycepin from *Cordyceps militaris*

**DOI:** 10.1016/j.jpha.2021.05.003

**Published:** 2021-07-02

**Authors:** Jian Chen, Hai-Fang Li, Guozhu Zhao, Jin-Ming Lin, Xiangwei He

**Affiliations:** a Beijing Advanced Innovation Center for Tree Breeding by Molecular Design, College of Biological Science and Technology, Beijing Forestry University, Beijing, 100083, China; b Department of Chemistry, Beijing Key Laboratory of Microanalytical Methods and Instrumentation, MOE Key Laboratory of Bioorganic Phosphorus Chemistry & Chemical Biology, Tsinghua University, Beijing, 100084, China

**Keywords:** Cordycepin, MALDI-MS, Isotope internal standard, Quantitative analysis, Liquid fermentation

## Abstract

Cordycepin, which has great immunomodulatory activities such as anticancer, antifungal, antivirus, antileukemia and lipid-lowering ones, is the secondary metabolite of *Cordyceps militaris* (*C. militaris*). Liquid submerged fermentation is the common cultivation process to produce cordycepin. To optimize the fermentation process and improve production, monitoring the cordycepin secretion in the fermentation is essential. The measurement based on chromatography-mass spectrometry methods is generally involved in the complex sample pretreatments and time-consuming separation, so more rapid and convenient methods are required. Matrix-assisted laser desorption ionization mass spectrometry (MALDI-MS) is more attractive for faster and direct detection. Therefore, MALDI-MS detection combined with isotope-labeled internal standard was applied to the measurement of cordycepin content in the fermentation broth and mycelium. This method made accurate quantification of cordycepin in the range of 5–400 μg/mL with a relative standard deviation of 5.6%. The recovery rates of fermentation samples after the 1, 13, and 25 days were 90.15%, 94.27%, and 95.06%, respectively. The contents of cordycepin in the mycelium and fermentation broth were 136 mg/g and 148.39 mg/mL on the 20th culture day, respectively. The cordycepin secretion curve of the liquid fermentation of *C. militaris* was real-time traced over 25 days.

## Highlights

• A rapid quantification method of cordycepin based on MALDI-MS is proposed.
• The quantification relies on the stable isotope standard method.
• Rapid determination of the cordycepin content in the liquid fermentation broth of *Cordyceps militaris* without pre-treatment.
• Monitoring the fermentation state of *C. militaris* fermentation broth is benefit to improve the yield of cordycepin.

## Graphical abstract

**Figure:**
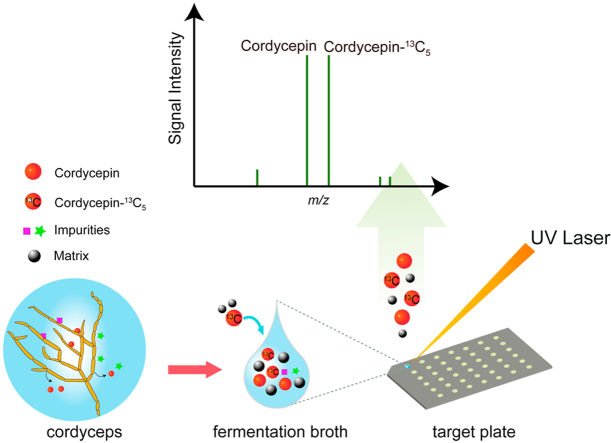

## 1 Introduction

*Cordyceps sinensis (C. sinensis)* is one type of valuable traditional Chinese medicine. The main active ingredient of *C. sinensis* is cordycepin (3′-deoxyadenosine) [1], which is a nucleoside analog and has immunomodulatory function, anticancer, antifungal, antivirus, antileukemia, lipid-lowering, hepatotoxicity-protecting and herbicidal properties [[2], [3], [4], [5], [6], [7], [8], [9]]. *C. sinensis* has very high commercial values due to its very low yield even by artificial cultivation [10]. The cordycepin produced from *Cordyceps militaris* (*C. militaris*) has pharmacological activities similar to those of natural *C. sinensis* [11]*,* while the cultivation cost of *C. militaris* is much lower than that of *C. sinensis*. Therefore, cordycepin from *C. militaris* becomes a perfect substitute healthy product for *C. sinensis* [12]*.*

Liquid submerged fermentation, one of the important methods to produce cordycepin [13], has the advantages of a short production period and good controllability. The real-time quantitative analysis of cordycepin is an important indicator to optimize the fermentation process and fermentation conditions. Capillary electrophoresis or high performance liquid chromatography coupling with mass spectrometry (HPLC-MS) is generally used for the detection of cordycepin [14,15]. Due to the complex components in the fermentation broth of *C. militaris*, some impurities interfere with the quantification of cordycepin. Therefore, solid-phase extraction is commonly required before HPLC-MS [16], which includes multi-step options and takes a long time for pretreatment. Matrix-assisted laser desorption ionization time-of-flight mass spectrometry (MALDI-TOF MS) is more attractive for faster and direct detection without requiring complex sample pretreatment because the solid plot ionization mode could tolerate complex and high salt samples. MALDI-MS has been widely used to identify biological macromolecules [17,18]. In recent years, some studies have applied it to the detection of small molecules with special matrices [19,20]. Due to the inhomogeneity of crystallization in MALDI-MS, it is difficult to obtain stable and reproducible signals. The stable isotope internal standard method makes it possible to realize quantitative detection with MALDI-MS. Our group has developed surface-assisted laser desorption ionization mass spectrometry based on gold nanoparticles for determining the ratio of glutathione to glutathione disulfide in cells in virtue of isotopic internal standard [21].

**Scheme 1:**
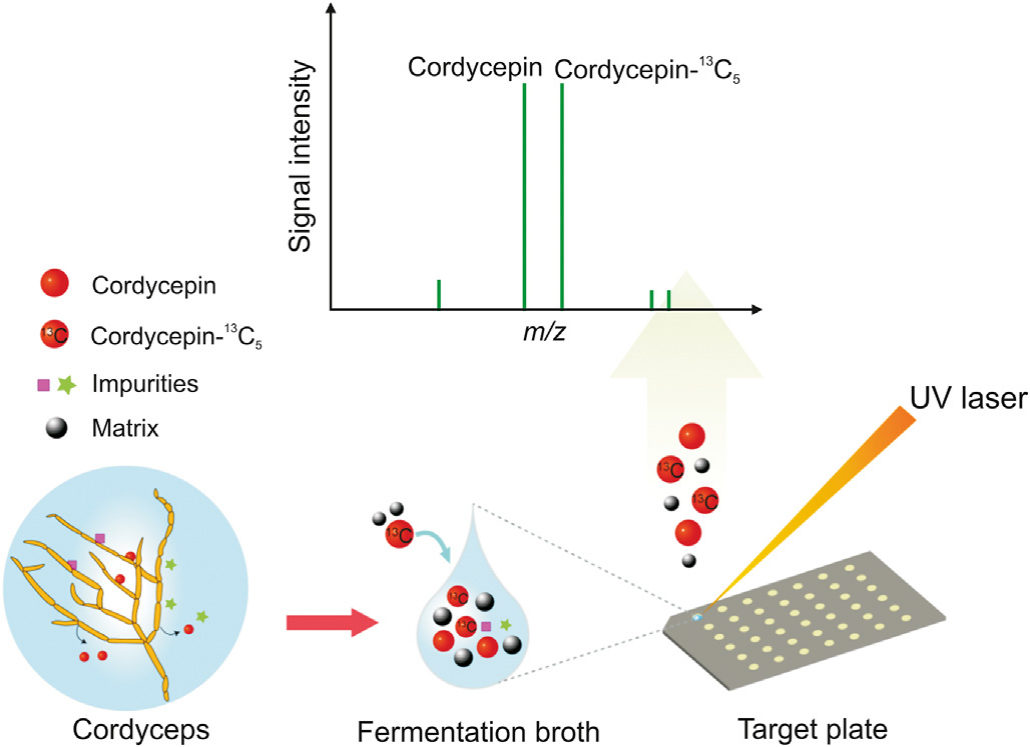
Schematic illustration of matrix-assisted laser desorption ionization mass spectrometry (MALDI-MS) quantitative analysis of secreted cordycepin by *C. militaris*.

In this paper, the rapid determination of cordycepin in the fermentation broth of *C. militaris* was carried out based on MALDI-MS. C-13 labeled cordycepin (cordycepin-^13^C_5_) was used as an internal standard to be added into the fermentation broth at a certain concentration for quantitative measurement of cordycepin (Scheme 1). When 2,5-dihydroxybenzoic acid (DHB) was the assisted matrix, a good quantitative linear relationship between cordycepin concentration and isotope ratio of cordycepin/cordycepin-^13^C_5_ could be obtained. With this quantitative method, the crude fermentation broth was directly detected without any pretreatment by MALDI-MS. The cordycepin secretion curve over 25 days was conveniently real-time traced to monitor the liquid fermentation process of *C. militaris*. Moreover, the cordycepin content in *C. militaris* mycelium was also determined after simple liquid extraction. Our quantitative analysis, which involved stages ranging from sample loading to getting test results, took less than 15 min.

## 2 Materials and methods

### 2.1 Materials

*C. militaris* was provided by State Key Lab of Mycology, Institute of Microbiology, Chinese Academy of Sciences (Beijing, China). Agar, dextrose, glucose, peptone, KH_2_PO_4_, MgSO_4_·7H_2_O, and vitamin B were purchased from Beijing Yixiubogu Biotech. Co., Ltd. (Beijing, China). Standard cordycepin-^13^C_5_ was purchased from Toronto Reserach Chemicals (Toronto, Canada). 2,5-dihydroxybenzoic acid (DHB), α-cyano-4-hydroxycinnamic acid (CHCA), and standard cordycepin were purchased from Sigma Aldrich (St. Louis, MO, USA).

### 2.2 Liquid fermentation of C. militaris

First, the *C. militaris* strains were inoculated on potato dextrose agar plates supplemented with 200 g/L (*m*/*V*) potato, 20 g/L dextrose, 16 g/L agar, 3 g/L peptone, 2 g/L KH_2_PO_4_, 20 g/L MgSO_4_, and 25 mg/L vitamin B at pH 6.5–7.0. The *C. militaris* were cultured in a biochemical incubator at 21 °C in the dark for 10 days. Fungal clumps of 1 cm^2^ were picked from the activated *C. militaris* species by using a sterile inoculating needle, and then inoculated in a flask containing 100 mL of seed liquid culture medium in a full-temperature incubator shaking 130 rpm/min at 26 °C for 84 h. Finally, it was inoculated on the fermentation medium containing 20 g/L glucose, 20 g/L fish peptone, 0.5 g/L KH_2_PO_4,_ and 0.5 g/L MgSO_4_. The inoculum amount was 4% (*V*/*V*) inoculated in an Erlenmeyer flask keeping pH value at 5.5, the culture was performed by shaking for the first 3 days and then standing for 22 days at room temperature.

### 2.3 Extraction of cordycepin from fermentation broth and mycelium

The mycelium of *C. militaris* was extracted from the fermentation broth, and after the fermentation broth was filtered out, 50 mg of the mycelium was weighed and put in a centrifuge tube. 25% methanol of 1 mL was added to the mycelium sample. The mixture was placed on a shaker for 1 h and then placed under ultrasonic treatment for 30 min. Finally, the mixture was centrifuged at 8,000 rpm for 10 min to get an extract. The supernatant was extracted and it was mixed with matrix and cordycepin-^13^C_5_ standard solution at a volume ratio of 1:2:1, before being detected by MALDI-MS.

### 2.4 MALDI-MS detection

Cordycepin content in the fermentation broth was detected on MALDI-TOF MS spectrometer (AXIMA Performance, Shimadzu Co., Ltd., Kyoto, Japan) with 337 nm nitrogen laser. DHB standard of 20 mg was dissolved in 1 mL acetonitrile:H_2_O (1:1, *V*/*V*) as matrix solution. The same concentration of CHCA matrix was prepared in acetonitrile/H_2_O (1:1, *V*/*V*) containing 0.1% (*V/V*) trifluoroacetic acid. One microliter of matrix solution and 1 μL of the sample were mixed and then spotted 0.5 μL on the stainless plate. The detection was operated in cation reflection mode and the *m/z* range was set from 100 to 500 Da. The cordycepin and cordycepin-^13^C_5_ standard were also detected by electrospray ionization ion trap/time of flight mass spectrometry (ESI-IT/TOF MS, Shimadzu, Co., Ltd., Kyoto, Japan).

For quantification, linearity was investigated by a stable isotope-labeled internal standard method. The cordycepin standard solutions were prepared at serial concentrations of 5, 20, 40, 100, 200, and 400 μg/mL with spiking 100 μg/mL of a cordycepin-^13^C_5_ internal standard. The MS peaks at *m/z* 252 and 274 were [^251^M+H]^+^ and [^251^M+Na]^+^ molecular ions of cordycepin, the peaks at *m/z* 257 and 279 were [^256^M+H]^+^ and [^256^M+Na]^+^ ions of cordycepin-^13^C_5_, respectively. The linear relationship between the concentration of cordycepin and the signal ratio of cordycepin ion to cordycepin-^13^C_5_ ion was obtained. According to the linear equation, the content of cordycepin can be calculated with the signal ratio of cordycepin ion to cordycepin-^13^C_5_.

Standard cordycepin was added to the fermentation broth for recovery analysis. The cordycepin recovery rates of initial, intermediate and terminate fermentation stages were concerned. The concentrations of standard cordycepin were at 5, 40 and 80 μg/mL levels in the 1, 13, and 25 days fermentation broth, respectively.

## 3 Results and discussion

### 3.1 Selection of MALDI matrix

**Fig. 1:**
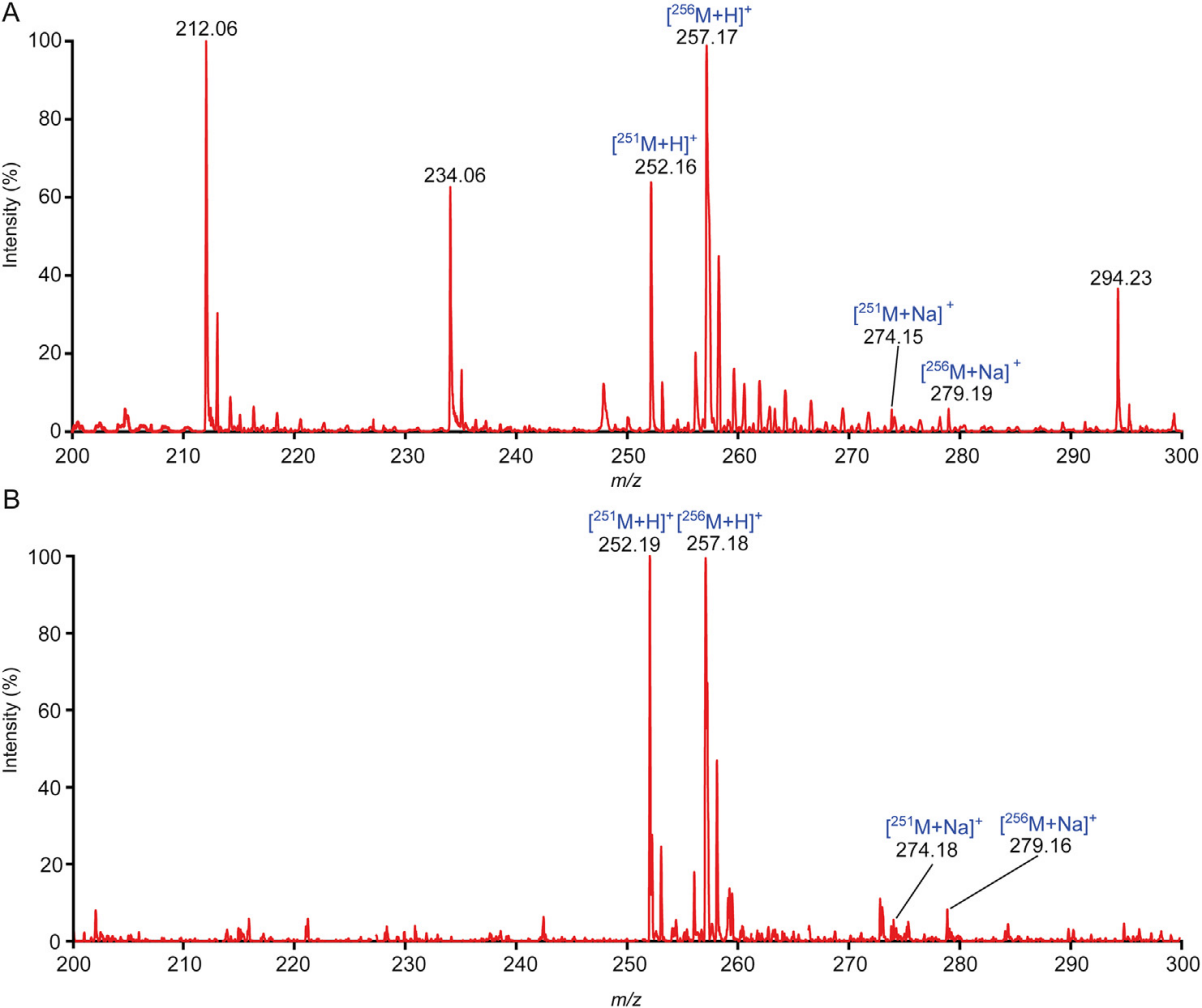
Mass spectra of cordycepin and cordycepin-^13^C_5_ standard assisted by different matrices. (A) α-cyano-4-hydroxycinnamic acid (CHCA) as matrix, (B) 2,5-dihydroxybenzoic acid (DHB) as matrix.

For detection of cordycepin, DHB and CHCA matrices commonly used for small molecules were tried [22]. The signal sensitivity and stability of DHB and CHCA as matrices were compared as shown in Fig. S1. The cordycepin molecular ion peaks of [^251^M+H]^+^ at *m/z* 252 and [^256^M+H]^+^ isotope at *m/z* 257 were observed clearly. But the signal intensities of the targets are very different at the same concentration, even the *m/z* 212 signal peak almost exceeded the intensity of the cordycepin (Fig. 1A). It shows that the CHCA had a great interference effect on the ionization and quantitative analysis of the target. When DHB was used as the matrix, the peak intensities [^251^M+H]^+^ at *m/z* 252 and [^256^M+H]^+^ at *m/z* 257 were the same (Fig. 1B), and it was perfectly reproducible. Compared with that of CHCA, the background signal of DHB was obviously lower in the range of *m/z* 200–300 Da. The ionization of the cordycepin and the cordycepin-^13^C_5_ was almost the same, due to their same chemical structure and chemical properties. And the distribution of target peaks signal intensity of target peaks was similar to that of ESI-MS (Fig. S2). Compared with DHB, CHCA brought higher background signals for detection of cordycepin. The complex background signals of CHCA affected the detection of small molecules as previouly reported [23]. In addition, it was presumed that DHB was a better proton donor than CHCA for assisted ionization, so the stronger signal could be obtained with DHB matrix. Hence, DHB is more suitable as a matrix for cordycepin quantitative detection on MALDI-MS.

### 3.2 Optimized cordycepin quantification

**Fig. 2:**
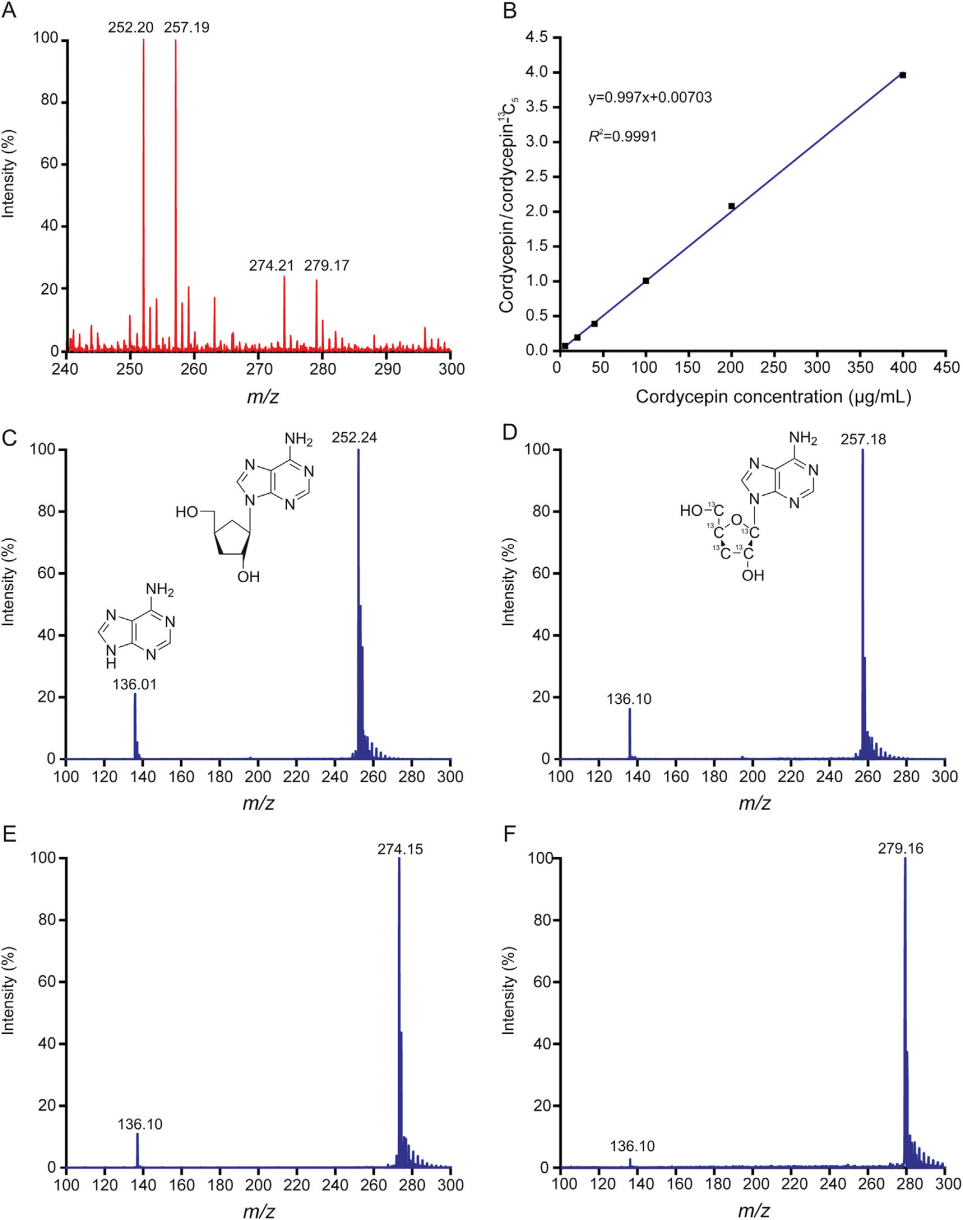
The correspondence relationship between the [M+H]^+^ and [M+Na]^+^ peaks intensity and MALDI quantitative analysis. (A) Mass spectra of the mixture of cordycepin and cordycepin-^13^C_5_ at the same concentration of 100 μg/mL in the blank fermentation medium. (B) Calibration curve between cordycepin content and the signal ratio of cordycepin to cordycepin-^13^C_5_. (C–F) Fragment mass spectra of [^251^M+H]^+^, [^256^M+H]^+^, [^251^M+Na]^+^ and [^256^M+Na]^+^, respectively.

The difficulties in direct detection of fermentation broth are mainly due to the interferences of macromolecules and salt ions in the crude fermentation broth. Analysis methods of gas chromatography-MS and HPLC-MS generally involve a complicated sample pretreatment process before detection [24,25]. To enable robust and rapid quantitative analysis of *C. militaris* fermentation broth, MALDI-MS by stable isotope internal standard was developed. The same structure and chemical properties of cordycepin and cordycepin-^13^C_5_ effectively eliminated the signal differences caused by the inhomogeneity of matrix crystallization. After cordycepin and cordycepin-^13^C_5_ of the same concentration were added to the fermentation medium, it was found that the strength of [^251^M+Na]^+^ and [^251^M+H]^+^ cordycepin peaks was almost the same as that of [^256^M+Na]^+^ and [^256^M+H]^+^ cordycepin-^13^C_5_ peaks (Fig. 2A). The same fragmentation of *m/z* 136 was obtained from the secondary ion of precursor ions of *m/z* 252, 257, 274, and 279. The *m/z* 136 fragment was the hydrogenation ion peaks of adenine, which was one of the precursor compounds for synthesis of cordycepin (Fig. 2C–F). Since *m/z* 274 and 279 were tested to be the sodium ion peaks of cordycepin and cordycepin-^13^C_5_, it is better to calculate the sum of [M+H]^+^ and [M+Na]^+^ peak intensities for quantitative analysis.

The linearity of the MALDI-MS detection for cordycepin was investigated in the concentrations ranging from 5 to 400 μg/mL, with a relative deviation of 5.6%. The stable isotope internal standard method enabled an excellent linear relationship between cordycepin concentration and the signal ratio of cordycepin to cordycepin-^13^C_5_ with a correlation coefficient of 0.9991 (Fig. 2B). The detection limit was 0.2 μg/mL (S/N = 3). And the recovery rates of fermentation samples after the 1, 13, and 25 days were 90.15%, 94.27%, and 95.06%, respectively. These results demonstrated the suitability of the isotope internal standard method and the capability of accurate quantitative analysis. The merits of this method lay not only in being free from the complex sample preparation but also being sensitive and rapid. For a batch of fermentation determination, it can be real-time and high-throughput detected. Our quantitative analysis took less than 15 min for each sample.

### 3.3 Cordycepin content in mycelium and fermentation broth

**Fig. 3:**
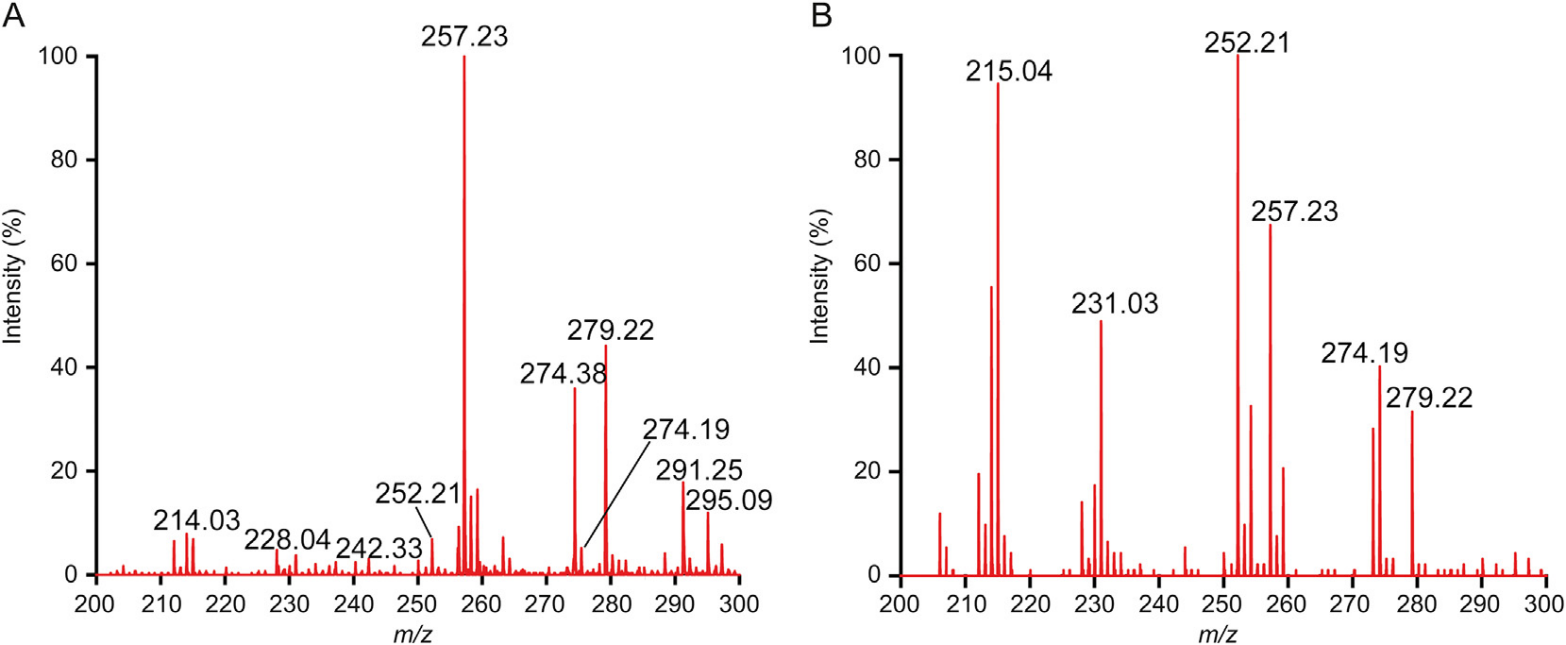
Comparison of MALDI-MS profiles of fermentation broth and mycelium on the 20th culture day, (A) mycelium extract and (B) fermentation broth spiked with 100 μg/mL internal standard.

It is important to determine the distribution of cordycepin in the fermentation broth or in the mycelium for termination of fermentation. Simultaneous detection of the mycelium and the fermentation broth at the same culture time was carried out. As shown in Fig. 3, both [M+H]^+^ and [M+Na]^+^ peaks were observed in the spectra of mycelium and fermentation broth on the 20th culture day. Since the linear equation was related to the sum of [M+H]^+^ and [M+Na]^+^, the target peaks intensities of [M+H]^+^ and [M+Na]^+^ were summed, and the content of cordycepin in the mycelium was calculated to be 136 μg/g, while the cordycepin concentration in the fermentation broth was 148.39 μg/mL. A certain amount of mycelium were produced in every 100 mL of fermentation broth (Fig. S3), but the dry weight of this mycelium is less than 5 g. Therefore, the cordycepin secreted into the fermentation broth was 20 times greater than that in the mycelium. Cordycepin, as a secondary metabolite of *C. militaris* [26], was produced by the mycelium tissue during the fermentation process and continuously secreted into the fermentation broth. It is meaningful to monitor the cordycepin content in fermentation broth for high yield.

**Fig. 4:**
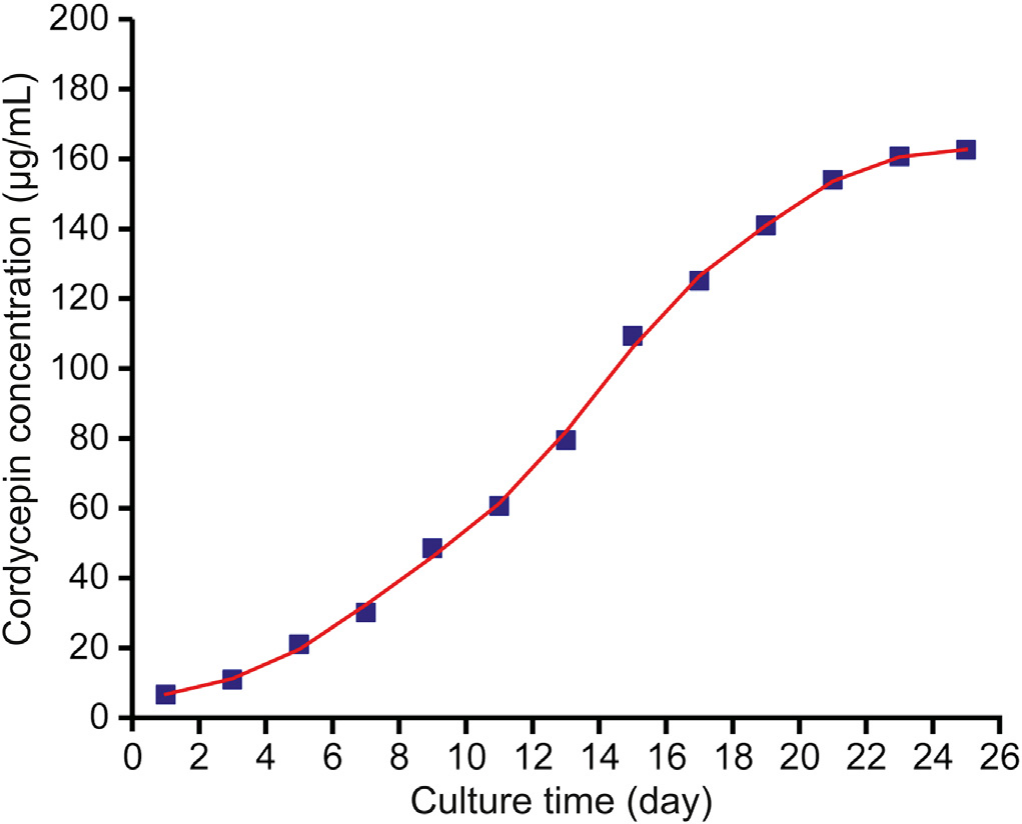
Secretion curve of cordycepin in *C. militaris* fermentation broth over 25 days.

The cordycepin secreted in the *C. militaris* fermentation broth during the fermentation process was determined. The curve of cordycepin secretion was obtained over 25 days (Fig. 4). The *C. militaris* speeded up cordycepin secretion from the start until to 20 days fermentation. Then cordycepin secretion slowed down due to the depletion of medium components and the increase of metabolic wastes. The real-time tracing of cordycepin secretion is of great significance for monitoring the production of cordycepin and determining the fastest production rate of cordycepin.

## 4 Conclusion

Monitoring the cordycepin secretion in liquid fermentation is essential for optimizing the fermentation process and improving production. In this study, quantitative MALDI-MS detection combined with the isotope-labeled internal standard for rapid determination of cordycepin secretion has been established. The isotope internal standard method supports an excellent linear relationship between cordycepin content and the signal ratio of cordycepin to cordycepin-^13^C_5_. The high recoveries of fermentation broth at three secretion stages over 90% were obtained. Based on the quantitative method, the cordycepin secretion curve of liquid fermentation of *C. militaris* was real-time traced over 25 days. The results indicated that the developed MALDI-MS analysis of cordycepin provided a simple and convenient way for monitoring the yield of *C. militaris* fermentation broth.

## Declaration of competing interest

The authors declare that there are no conflicts of interest.

## References

[1] Chamyuang S., Owatworakit A., Honda Y. (2019). New insights into cordycepin production in Cordyceps militaris and applications. Ann. Transl. Med..

[2] De Silva D.D., Rapior S., Fons F. (2012). Medicinal mushrooms in supportive cancer therapies: an approach to anti-cancer effects and putative mechanisms of action. Fungal Divers..

[3] Yoshikawa N., Yamada S., Takeuchi C. (2008). Cordycepin (3’-deoxyadenosine) inhibits the growth of B16-BL6 mouse melanoma cells through the stimulation of adenosine A3 receptor followed by glycogen synthase kinase-3βactivation and cyclin D1 suppression. Arch. Pharmacol..

[4] Sugar A.M., Mc Caffrey R.P. (1998). Antifungal activity of 3'-deoxyadenosine (cordycepin). Antimicrob. Agents Chemother..

[5] Hashimoto K., Simizu B. (1976). Effect of cordycepin on the replication of western equine encephalitis virus. Arch. Virol..

[6] De Silva D.D., Rapior S., Sudarman E. (2013). Bioactive metabolites from macrofungi: ethnopharmacology, biological activities and chemistry. Fungal Divers..

[7] De Silva D.D., Rapior S., Hyde K.D. (2012). Medicinal mushrooms in prevention and control of diabetes mellitus. Fungal Divers..

[8] Cha J.Y., Ahn H.Y., Cho Y.S. (2013). Protective effect of cordycepin-enriched Cordyceps militaris on alcoholic hepatotoxicity in Sprague-Dawley rats. Food Chem. Toxicol..

[9] Quy T.N., Xuan T.D., Andriana Y. (2019). Cordycepin isolated from Cordyceps militaris: its newly discovered herbicidal property and potential plant-based novel alternative to glyphosate. Molecules.

[10] Li X., Liu Q., Li W. (2018). A breakthrough in the artificial cultivation of Chinese cordyceps on a large-scale and its impact on science, the economy, and industry. Crit. Rev. Biotechnol..

[11] Yin J., Xin X.D., Weng Y.J. (2018). Genotypic analysis of degenerative Cordyceps militaris cultured in the pupa of Bombyx mori. Entomol. Res..

[12] Wang F., Song X., Dong X.H. (2017). DASH-type cryptochromes regulate fruiting body development and secondary metabolism differently than CmWC-1 in the fungus Cordyceps militaris. Biotechnology.

[13] Yan J.K., Wang W.Q., Wu J.Y. (2014). Recent advances in Cordyceps sinensis polysaccharides: mycelial fermentation, isolation, structure, and bioactivities: a review. J. Funct. Foods.

[14] Yang F.Q., Ge L.Y., Yong J.W. (2009). Determination of nucleosides and nucleobases in different species of Cordyceps by capillary electrophoresis-mass spectrometry. J. Pharmaceut. Biomed. Anal..

[15] Hu H.K., Xiao L., Zheng B.G. (2015). Identification of chemical markers in Cordyceps sinensis by HPLC-MS/MS. Anal. Bioanal. Chem..

[16] Wang Z.B., Li N., Wang M. (2013). Simultaneous determination of nucleosides and their bases in Cordyceps sinensis and its substitutes by matrix solid-phase dispersion extraction and HPLC. J. Separ. Sci..

[17] Karas M., Hillenkamp F. (1988). Laser desorption ionization of proteins with molecular masses exceeding 10,000 daltons. Anal. Chem..

[18] Castro J.A., Köster C., Wilkins C. (2010). Matrix-assisted laser desorption/ionization of high-mass molecules by Fourier-transform mass spectrometry. Rapid Commun. Mass Spectrom..

[19] Tang H.Z., Ma Y.L., Liu F. (2017). Detection of small molecules using SBA-15 modified CHCA as a novel matrix of MALDI-TOF MS. Int. J. Mass Spectrom..

[20] Zhao F., Li Y.Q., Wang J. (2018). Dual-ion-mode MALDI MS detection of small molecules with the O-P, N doped carbon/graphene matrix. ACS Appl. Mater. Interfaces.

[21] Li M., Mao S.F., Wang S.Q. (2019). Chip-based SALDI-MS for rapid determination of intracellular ratios of glutathione to glutathione disulfide. Sci. China Chem..

[22] Dong J.L., Ning W.J., Mans D.J. (2018). A binary matrix for the rapid detection and characterization of small-molecule cardiovascular drugs by MALDI-MS and MS/MS. Anal. Methods.

[23] Catharino R.R., Marques L.D.A., Santos L.S. (2005). Aflatoxin screening by MALDI-TOF mass spectrometry. Anal. Chem..

[24] Zhang H.Y., Li Y.H., Mi J.N. (2017). GC-MS profiling of volatile components in different fermentation products of Cordyceps sinensis mycelia. Molecules.

[25] Cheng Y.H., Hsieh Y.C., Yu Y.H. (2019). Effect of Cordyceps militaris hot water extract on immunomodulation-associated gene expression in broilers, Gallus gallus. J. Poultry Sci..

[26] Hu D., Chen Y., Sun C.H. (2018). Genome guided investigation of antibiotics producing actinomycetales strain isolated from a Macau mangrove ecosystem. Sci. Rep..

