# Supplementary material for: Matrix-assisted laser desorption ionization mass spectrometry based quantitative analysis of cordycepin from Cordyceps militaris
Source: J Pharm Anal. 2021 Jul 2;11(4):499–504. doi: 10.1016/j.jpha.2021.05.003 (PMC8424359; doi:10.1016/j.jpha.2021.05.003)
Supplement: Multimedia component 1 [file mmc1.docx]

**Supplementary Information**

MALDI-MS Based Quantitative Analysis of Cordycepin from Cordyceps Militaris

Jian Chen ^a, b^, Hai-Fang Li ^b, *^, Guozhu Zhao ^a^, Jin-Ming Lin ^b^, Xiangwei He ^a, *^

^a^ Beijing Advanced Innovation Center for Tree Breeding by Molecular Design, College of Biological Science and Technology, Beijing Forestry University, Beijing 100083, China

^b^ Department of Chemistry, Beijing Key Laboratory of Microanalytical Methods and Instrumentation, MOE Key Laboratory of Bioorganic Phosphorus Chemistry & Chemical Biology, Tsinghua University, Beijing, 100084, China

*Corresponding authors:

E-mail: [hexiangwei@bjfu.edu.cn](mailto:hexiangwei@bjfu.edu.cn)

Tel.: 86-10-62336016

E-mail: lihaifang@mail.tsinghua.edu.cn

Tel.: 86-10-62797463; Fax: 86-10-62797463

**Supplementary Figures**


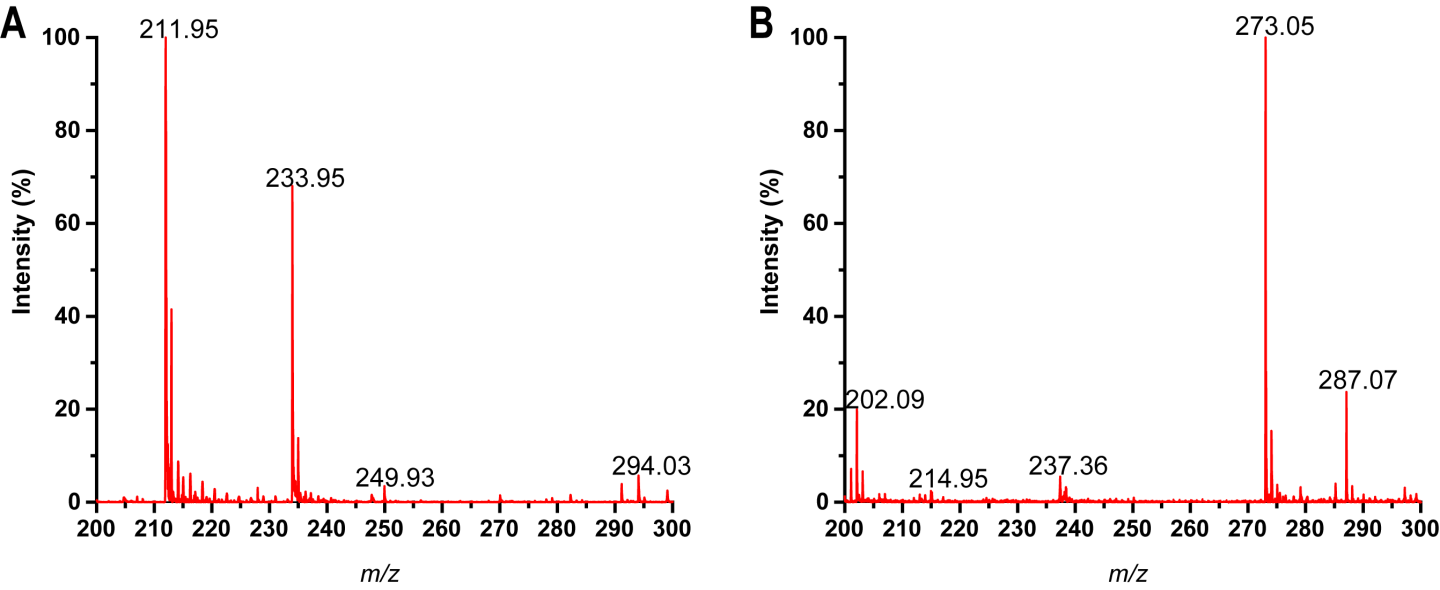


**Fig.S1** Comparison of matrix-assisted laser desorption ionization mass spectrometry (MALDI-MS) profiles of two matrices. (A) α-cyano-4-hydroxycinnamic acid (CHCA) background. (B) 2,5-dihydroxybenzoic acid (DHB) background.


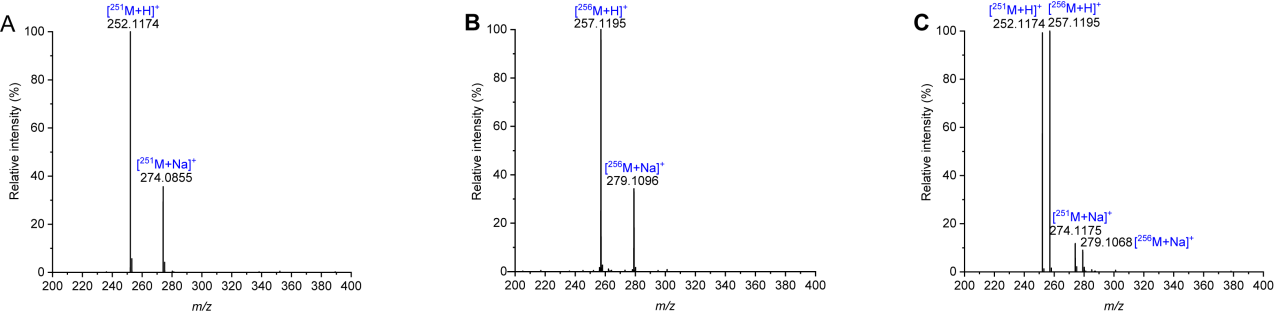


**Fig.S2** Electrospray ionization ion trap/time of flight mass spectrometry (ESI-IT/TOF MS) profiles of cordycepin and cordycepin-^13^C_5_ standrand. (A) Cordycepin standard (B) Cordycepin-^13^C_5_ standard. (C) Cordycepin standard with cordycepin-^13^C_5_ standard at same concentration.


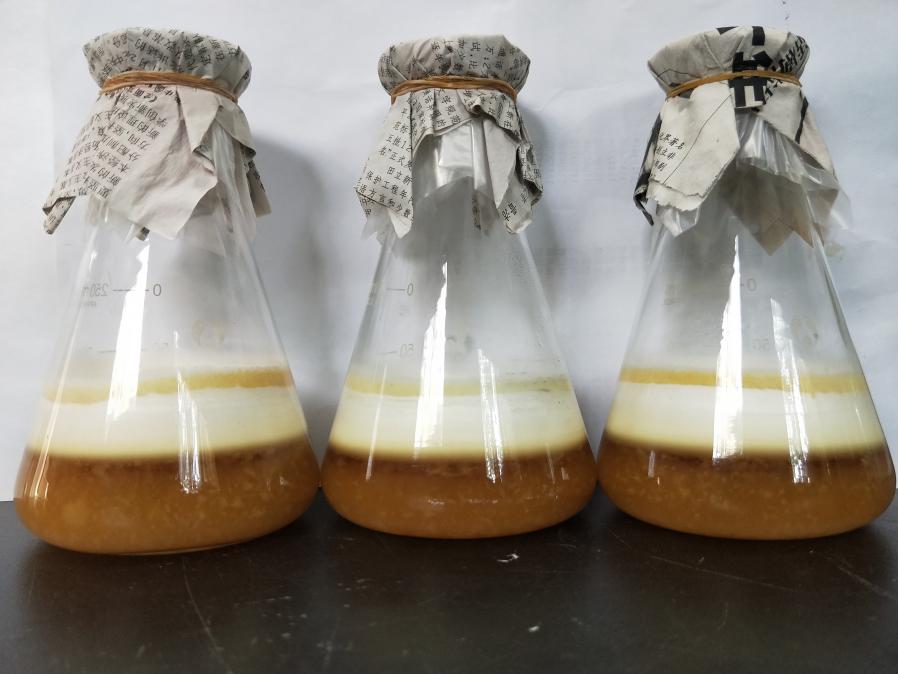


**Fig.S3** Fermentation broth containing mycelium after 20 days of culture.
